# Genetic information supports a causal relationship between trace elements, inflammatory proteins, and COPD: evidence from a Mendelian randomization analysis

**DOI:** 10.3389/fnut.2024.1430606

**Published:** 2024-08-14

**Authors:** Zhenghua Cao, Shengkun Zhao, Tong Wu, Feng Sun, Huan Ding, Shaodan Hu, Li Shi

**Affiliations:** ^1^Graduate School, Changchun University of Traditional Chinese Medicine, Changchun, Jilin, China; ^2^Geriatric Department, Suzhou Hospital of Integrated Traditional Chinese and Western Medicine, Suzhou, Jiangsu, China; ^3^Respiratory Disease Department, Affiliated Hospital of Changchun University of Traditional Chinese Medicine, Changchun, Jilin, China

**Keywords:** trace elements, inflammatory proteins, COPD, Mendelian randomization, mediation analysis

## Abstract

**Objective:**

Dietary factors and nutritional status may be among the risk factors for Chronic Obstructive Pulmonary Disease (COPD). There exists a certain correlation between trace elements and COPD. Through Mendelian Randomization (MR) analysis, we investigated the causal relationships between trace elements, inflammatory proteins, and COPD.

**Methods:**

We employed MR, multivariable MR (MVMR), and two-step MR (TSMR) approaches to assess the causal links between 15 trace elements and COPD, with 91 inflammatory proteins serving as mediators to further elucidate the tripartite causal relationships.

**Results:**

Trace elements such as Folate (OR = 1.293, 95%CI 1.027–1.628; *p* = 0.029), Vitamin D (OR = 1.331, 95%CI 1.071–1.654; *p* = 0.010), Vitamin B12 (OR = 1.424, 95%CI 1.108–1.828; *p* = 0.006), and Iron (OR = 0.741, 95%CI 0.580–0.946; *p* = 0.016) demonstrated causal relationships with COPD. No causal relationship was observed in reverse MR. After adjusting for BMI, Folate (OR = 1.633, 95%CI 1.098–2.429; *p* = 0.015), Iron (OR = 0.507, 95%CI 0.31–0.778; *p* = 0.001), and Vitamin D (OR = 1.511, 95%CI 1.029–2.217; *p* = 0.034) were identified as independent risk factors for COPD, whereas Vitamin B12 (OR = 1.118, 95%CI 0.751–1.666; *p* = 0.581) was not. Mediation analysis indicated that CDCP1 (5.76%) may play a mediating role between Iron and COPD.

**Conclusion:**

Trace elements such as Folate, Vitamin D, Vitamin B12, and Iron have causal relationships with COPD. After BMI adjustment, Folate, Vitamin D, and Iron emerge as independent risk factors. Furthermore, the inflammatory protein CDCP1 may partially mediate the causal relationship between Iron and COPD, offering a scientific basis for dietary recommendations that could benefit COPD patients. The supplementation of trace elements may be advantageous for individuals suffering from COPD.

## 1 Introduction

Chronic Obstructive Pulmonary Disease (COPD) is a heterogeneous ailment that is progressively becoming the third leading cause of death globally (1). It is primarily characterized by airway pathologies (bronchitis, bronchiolitis) and/or alveolar abnormalities (emphysema) leading to chronic respiratory symptoms (dyspnea, cough, expectoration) and a persistent, progressive limitation of airflow (2). Studies have revealed that nearly half of COPD patients experience weight loss (3) and diminished appetite (4), often resulting in an intake of trace elements significantly below the recommended dietary allowances (5). Observational studies have identified that malnutrition and weight loss are prevalent among COPD outpatient attendees (6), and nutritional supplementation can enhance the quality of life for these patients (7). Trace elements play a protective role in lung function, potentially decelerating the rate of pulmonary decline (8). They also influence the diffusing capacity of the lungs and the strength of the respiratory muscles (9). Deficiencies in trace elements are common in COPD and may influence the progression of the disease (10). Dietary interventions and targeted supplementation of single or multiple trace elements could be beneficial for patients with COPD (11).

Dietary factors and nutritional status may be among the risk factors for COPD. Alterations in dietary habits can modulate the impact of adverse environmental exposures on the lungs (12). For instance, excessive consumption of processed red meat has been associated with an increased risk of developing COPD (13), whereas a high dietary fiber intake is inversely related to the risk of COPD (14). Malnutrition can heighten the risk of mortality in patients with COPD (15), underscoring the pivotal role that nutrition plays in respiratory diseases (16). Relevant studies have identified that diet can influence the development of COPD through three primary mechanisms, with the most significant being the modulation of inflammation (17). Inflammatory responses are correlated with various diseases (18–20), and the intake of trace elements can alleviate the inflammatory reactions associated with COPD (21). Metal ions such as iron and copper in trace elements are crucial to the presence of pulmonary inflammation and oxidative stress in COPD (22), potentially leading to diminished activity of macrophages (23). Exposure to environments like iron factories increases the risk of COPD (24), whereas improving environmental risks can decrease it (25). Inhibiting ferroptosis may alleviate emphysema and airway inflammation (26). There is a correlation between copper and pulmonary inflammation (27). Zinc can mitigate the progression of COPD induced by harmful gasses and offers protective benefits to lung tissue (28). There is also a correlation between zinc and the pathogenesis of COPD (29). Supplementing with vitamins A and K may reduce the risk of emphysema (30), with vitamin K potentially improving the condition (31). Carotene is correlated with lung function (32) and may enhance pulmonary health (33). Vitamin D is associated with respiratory diseases (34), and vitamin E can reduce the risk of COPD (35). Thus, trace elements may be significant influencing factors for patients with COPD (36).

Although observational studies and systematic reviews have established a connection between trace elements, nutritional status (37–41), and COPD, suggesting that malnutrition and deficiencies in trace elements can adversely affect COPD patients, the precise causal relationships and underlying mechanisms remain unclear. Mendelian randomization (MR) is a potential method for causal inference, used to estimate the causal effects of exposure factors on outcomes while controlling for confounding factors and avoiding reverse causation (42). Therefore, we aim to utilize MR analysis to elucidate the causal relationships between trace elements, inflammatory factors, and COPD, thereby providing scientifically sound dietary recommendations for COPD patients.

## 2 Methods

### 2.1 Study design

This study employs MR analysis, focusing on 15 trace elements, including Copper, Calcium, Folate, Iron, Vitamin D, and Vitamin B6, as the primary exposures, with COPD as the outcome. To further explore the mechanisms underlying the causal relationship between trace elements and COPD, we consider 91 inflammatory proteins as potential mediators to determine whether these proteins play a significant mediating role in the causal pathway between trace elements and COPD. This research adheres to the guidelines of the Strengthening the Reporting of Observational Studies in Epidemiology using Mendelian Randomization (STROBE-MR) Statement (43).

**Figure 1:**
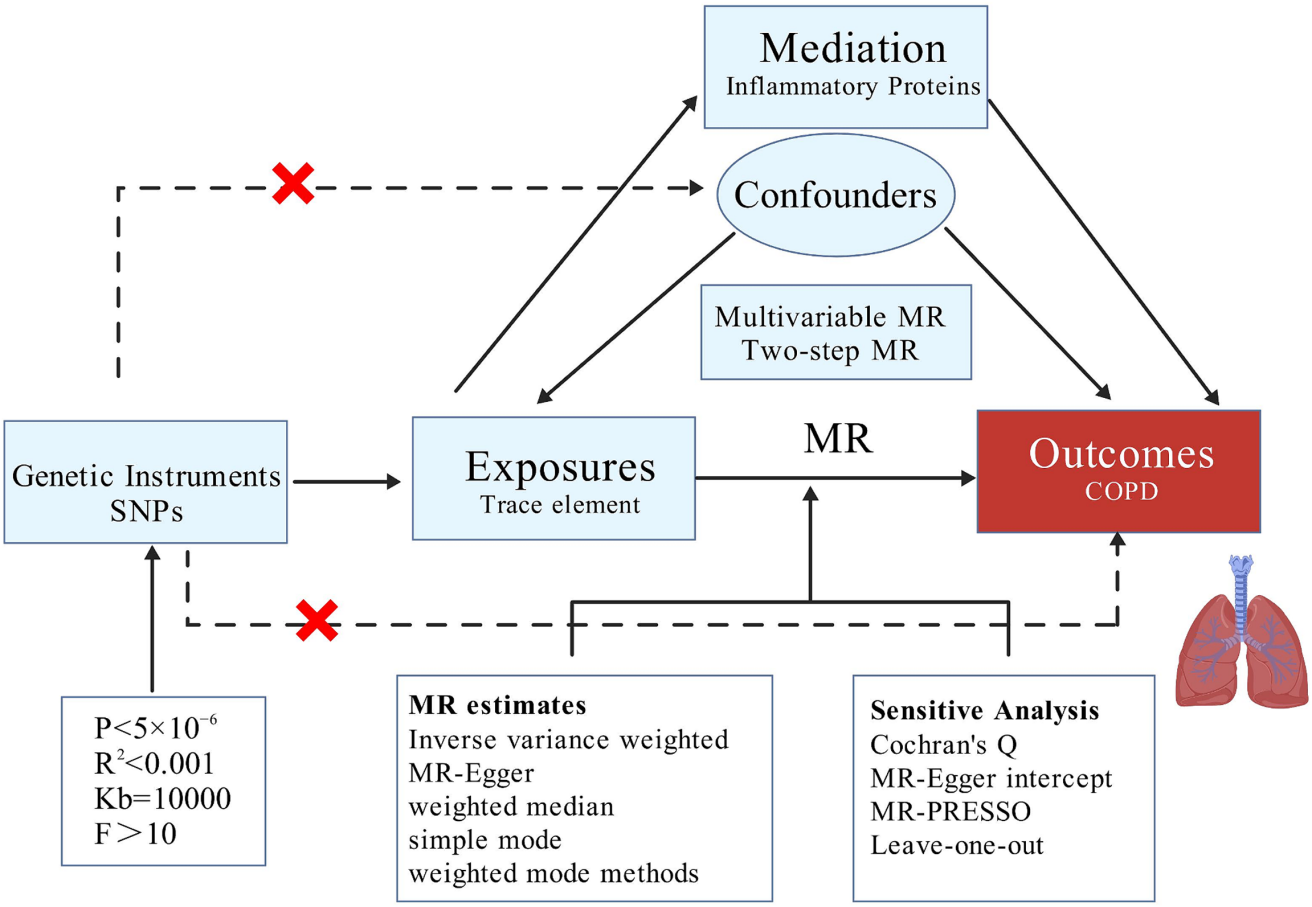
Research ideas.

Our MR analysis is structured into three distinct phases. Initially, we employ a two-sample MR approach to investigate whether a causal relationship exists between trace elements and COPD, and to ascertain the presence of any reverse causality, thereby determining the feasibility of further mediation analysis. Subsequently, after adjusting for BMI, we conduct MVMR to identify which trace elements independently contribute to risk. Lastly, we utilize TSMR to examine whether the causal effects are mediated by any of the 91 inflammatory proteins, thus performing mediation analysis and elucidating the proportion of the mediation effect (Figure 1).

### 2.2 Data sources

The genetic information for the 15 trace elements is sourced from the GWAS database,^1^ all pertaining to European populations. The data for the 91 inflammatory proteins are derived from a 2023 study involving 14,824 Europeans (44), cataloged under the identifiers GCST90274758 to GCST90274848. The COPD data is obtained from the tenth round of analysis by the FinnGen database (45),^2^ also concerning European populations. Additionally, the genetic information for BMI is acquired from the GWAS database and is likewise representative of European demographics (Table 1).

**Table 1:** Genetic information data sources.

Name	Number	Samples	SNP
Copper	ieu-a-1073	2,603	2,543,646
Calcium	ukb-b-8951	64,979	9,851,867
Carotene	ukb-b-16202	64,979	9,851,867
Folate	ukb-b-11349	64,979	9,851,867
Iron	ukb-b-20447	64,979	9,851,867
Magnesium	ukb-b-7372	64,979	9,851,867
Potassium	ukb-b-17881	64,979	9,851,867
Selenium	ieu-a-1077	2,603	2,543,617
Vitamin A	ukb-b-9596	460,351	9,851,867
Vitamin B12	ukb-b-19524	64,979	9,851,867
Vitamin B6	ukb-b-7864	64,979	9,851,867
Vitamin C	ukb-b-19390	64,979	9,851,867
Vitamin D	ukb-b-18593	64,979	9,851,867
Vitamin E	ukb-b-6888	64,979	9,851,867
Zinc	ieu-a-1079	2,603	2,543,646
COPD	finngen_R10_J10_COPD	358,369	1,048,576
BMI	ieu-a-1089	120,286	8,654,252

### 2.3 Instrumental variable selection

The selection of instrumental variables must satisfy several assumptions (46): the instrumental variables should be closely associated with trace elements, independent of confounding factors in the exposure-outcome relationship, and must influence COPD solely through the trace elements (47). To ensure their relevance (48), we conduct an association analysis on the 15 trace elements using a significance threshold of *p* < 5 × 10^−6^. Subsequently, we eliminate any single nucleotide polymorphisms (SNPs) exhibiting linkage disequilibrium by applying criteria of *R*^2^ < 0.001 and Kb = 10,000 (49). We then calculate the F-statistic for the selected SNPs to exclude weak instrumental variables, considering an *F*-value greater than 10 as indicative of the absence of weak instrumental variables (50, 51).

### 2.4 Statistical analysis

We employed five methods to assess causality: Inverse Variance Weighted (IVW), MR-Egger, Weighted Median, Simple Mode, and Weighted Mode, with IVW serving as the primary method (47, 52). A *p*-value less than 0.05 indicates a causal relationship (53), while the other four methods serve as supplementary approaches (54). To evaluate the robustness of our results, we conducted sensitivity analysis using the “leave-one-out” technique (55). Additionally, we employed Cochran’s Q test, MR-Egger intercept test, and MR-PRESSO to test for pleiotropy and heterogeneity (56, 57), with a *p*-value greater than 0.05 indicating the absence of both (58, 59). Using the TSMR approach, we first calculated the total effect (β0) of trace elements on COPD, the effect of trace elements on inflammatory proteins (β1), and the effect of inflammatory proteins on COPD (β2). The mediating effect was computed as β1*β2, and the direct effect as the total effect minus the mediating effect. The proportion mediated was calculated as (β1 × β2)/β0 (60). All analyses were conducted using the R language (version 4.3.3). The specific package employed was TwoSampleMR (version 0.6.0).

## 3 Results

### 3.1 Causal relationship between 15 trace elements and COPD

Through the judicious selection of instrumental variables, we conducted an associative analysis, eliminated linkage disequilibrium and weak instrumental variables, and identified 188 SNPs across 15 trace elements, with the smallest F-statistic being 20.86 and the largest 84.68. Univariate MR analysis supports a causal relationship between trace elements such as Folate, Vitamin D, Vitamin B12, and Iron, and COPD. The results of the IVW analysis indicate a positive correlation between Folate (OR = 1.293, 95% CI 1.027–1.628; *p* = 0.029), Vitamin D (OR = 1.331, 95% CI 1.071–1.654; *p* = 0.010), and Vitamin B12 (OR = 1.424, 95% CI 1.108–1.828; *p* = 0.006) with COPD, while Iron shows a negative correlation (OR = 0.741, 95% CI 0.580–0.946; *p* = 0.016). Concurrently, reverse MR analysis revealed no reverse causality between Folate, Vitamin D, Vitamin B12, and Iron with COPD (*p* > 0.05).

**Figure 2:**
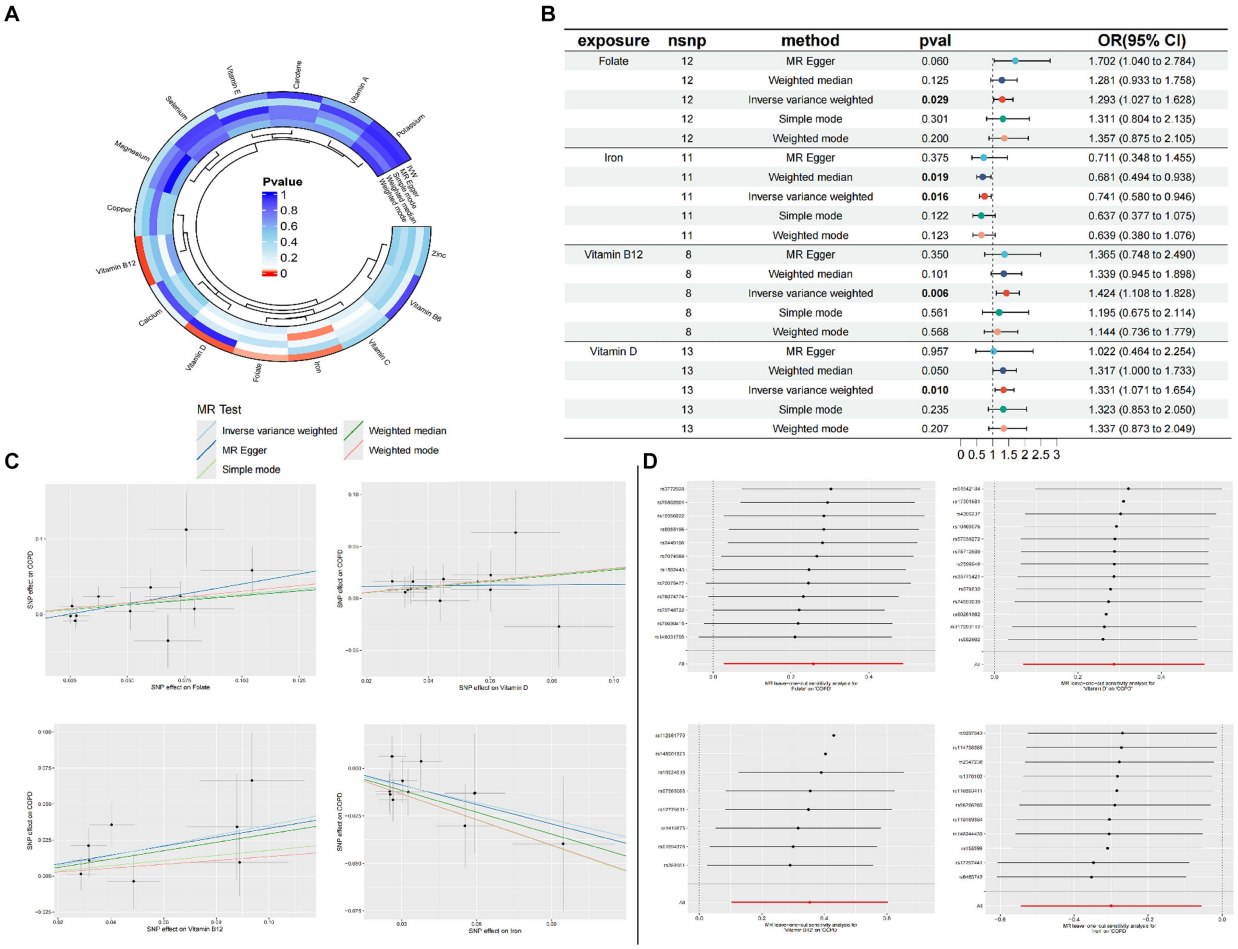
Circle plots of the five Mendelian randomization methods (*p* < 0.05) **(A)**; Forest plot of MR Analysis of trace elements and COPD **(B)**; MR scatter plot of trace elements and COPD **(C)**; Result of leave-one-out sensitivity analysis of trace elements and COPD **(D)**.

To evaluate the robustness of our analytical results, we employed Cochran’s Q test, the MR-Egger intercept test, and MR-PRESSO to examine pleiotropy and heterogeneity. No evidence of pleiotropy or heterogeneity was detected (*p* > 0.05). The leave-one-out analysis indicated that the exclusion of any single SNP would not significantly affect the estimation of causal relationships, suggesting that the results of the MR analysis are robust (Table 2; Figure 2).

**Table 2:** MR and sensitivity analyses of trace elements and COPD.

Exposure	Method	snp	Beta	Se	*p*	Pleiotropy test		Heterogeneity test	
						MR-PRESSO	MR-Egger intercept	IVW Q	MR-Egger Q
Folate	IVW	12	0.257	0.117	0.029	0.412	−0.012 (*p* = 0.247)	12.522 (*p* = 0.326)	10.879 (*p* = 0.367)
Vitamin D	IVW	13	0.286	0.111	0.010	0.974	0.011 (*p* = 0.510)	4.625 (*p* = 0.969)	4.161 (*p* = 0.965)
Vitamin B12	IVW	8	0.353	0.128	0.006	0.536	0.002 (*p* = 0.885)	6.161 (*p* = 0.521)	6.137 (*p* = 0.408)
Iron	IVW	11	−0.300	0.125	0.016	0.924	0.001 (*p* = 0.908)	4.497 (*p* = 0.922)	4.482 (*p* = 0.877)

### 3.2 Multivariate MR analysis

According to the results of the univariate MR analysis, a causal relationship exists between Folate, Vitamin D, Vitamin B12, and Iron with COPD. By adjusting for the influence of Body Mass Index (BMI), we conducted a MVMR analysis with these four trace elements and BMI. We discovered that the causal relationships with COPD persist for Folate (OR = 1.633, 95% CI 1.098–2.429; *p* = 0.015), Iron (OR = 0.507, 95% CI 0.31–0.778; *p* = 0.001), and Vitamin D (OR = 1.511, 95% CI 1.029–2.217; *p* = 0.034), indicating that Folate, Vitamin D, and Iron are independent risk factors for COPD. However, Vitamin B12 (OR = 1.118, 95% CI 0.751–1.666; *p* = 0.581) is not an independent risk factor for COPD (Table 3).

**Table 3:** MVMR and sensitivity analysis of trace elements and COPD.

Exposure	Outcome	Beta	Se	*p*	OR	95%CI	*Q*	Egger intercept	Ple	Het
Folate	COPD	0.491	0.202	0.015	1.633	1.098–2.429	41.396	−0.001	0.649	0.497
Vitamin D		0.418	0.195	0.034	1.511	1.029–2.217				
Vitamin B12		0.112	0.203	0.581	1.118	0.751–1.666				
Iron		−0.678	0.218	0.001	0.507	0.331–0.778				
BMI		−0.023	0.059	0.692	0.976	0.869–1.097				

Ple, Pleiotropy Test; Het, Heterogeneity Test.

### 3.3 TSMR and mediation analyses

We conducted a TSMR analysis, selecting 91 inflammatory proteins as instrumental variables. After analyzing associations, removing linkage disequilibrium, and excluding weak instrumental variables, we obtained 2,973 SNPs with the smallest F-statistic being 19.51 and the largest 1472.73. The univariate MR analysis of these 91 inflammatory proteins with COPD revealed positive causal relationships for CXCL10 (OR = 1.093, 95% CI 1.034–1.155; *p* = 0.001), EN-RAGE (OR = 1.117, 95% CI 1.041–1.198; *p* = 0.002), CD6 (OR = 1.064, 95% CI 1.022–1.107; *p* = 0.002), STAMPB (OR = 1.104, 95% CI 1.012–1.205; *p* = 0.025), and CXCL6 (OR = 1.062, 95% CI 1.015–1.112; *p* = 0.008). Conversely, negative causal relationships were observed for CD40 (OR = 0.948, 95% CI 0.903–0.997; *p* = 0.038) and CDCP1 (OR = 0.940, 95% CI 0.899–0.982; *p* = 0.006). Tests for pleiotropy and heterogeneity were conducted (*p* > 0.05), with consistent OR directions, and the leave-one-out analysis confirmed the robustness of the MR results (Table 4).

**Table 4:** MR and sensitivity analyses of inflammatory proteins and COPD.

Exposure	Method	snp	Beta	Se	*p*	Pleiotropy Test		Heterogeneity Test	
						MR-PRESSO	MR-Egger intercept	IVW Q	MR-Egger Q
CXCL10	IVW	33	0.089	0.028	0.001	0.650	−0.001 (*p* = 0.759)	28.273 (*p* = 0.655)	29.177 (*p* = 0.611)
EN-RAGE	IVW	23	0.111	0.036	0.002	0.288	−0.009 (*p* = 0.228)	26.450 (*p* = 0.232)	24.643 (*p* = 0.262)
CD6	IVW	25	0.062	0.020	0.002	0.764	−0.001 (*p* = 0.729)	20.113 (*p* = 0.690)	19.990 (*p* = 0.642)
CD40	IVW	23	−0.052	0.025	0.038	0.286	0.006 (*p* = 0.232)	20.664 (*p* = 0.224)	24.876 (*p* = 0.252)
CDCP1	IVW	36	−0.062	0.022	0.006	0.442	0.001 (*p* = 0.703)	36.182 (*p* = 0.413)	36.025 (*p* = 0.373)
STAMPB	IVW	20	0.099	0.045	0.025	0.282	−0.005 (*p* = 0.643)	22.473 (*p* = 0.261)	22.199 (*p* = 0.223)
CXCL6	IVW	22	0.061	0.023	0.008	0.604	0.004 (*p* = 0.367)	20.111 (*p* = 0.514)	19.261 (*p* = 0.504)

In further MR analyses of four trace elements and inflammatory proteins, we found positive correlations between Iron and CDCP1 (OR = 1.321, 95% CI 1.026–1.702; *p* = 0.031), as well as Iron and CXCL10 (OR = 1.389, 95% CI 1.070–1.803; *p* = 0.013). Conversely, negative correlations were observed between Folate and EN-RAGE (OR = 0.750, 95% CI 0.583–0.964; *p* = 0.025), and between Vitamin D (OR = 0.724, 95% CI 0.563–0.930; *p* = 0.011) and EN-RAGE (Table 5).

**Table 5:** MR and sensitivity analysis of trace elements and inflammatory proteins.

Exposure	Outcome	Method	snp	Beta	Se	*p*	Pleiotropy test		Heterogeneity test	
							MR-PRESSO	MR-Egger intercept	IVW Q	MR-Egger Q
Folate	EN-RAGE	IVW	13	−0.287	0.128	0.025	0.436	0.001 (*p* = 0.954)	12.387 (*p* = 0.415)	12.384 (*p* = 0.335)
Vitamin D	EN-RAGE	IVW	13	−0.322	0.127	0.011	0.936	−0.002 (*p* = 0.882)	5.738 (*p* = 0.928)	5.715 (*p* = 0.891)
Iron	CDCP1	IVW	12	0.278	0.129	0.031	0.697	−0.012 (*p* = 0.394)	8.208 (*p* = 0.694)	7.418 (*p* = 0.685)
Iron	CXCL10	IVW	12	0.328	0.133	0.013	0.575	−0.005 (*p* = 0.688)	9.596 (*p* = 0.566)	9.426 (*p* = 0.492)

**Figure 3:**
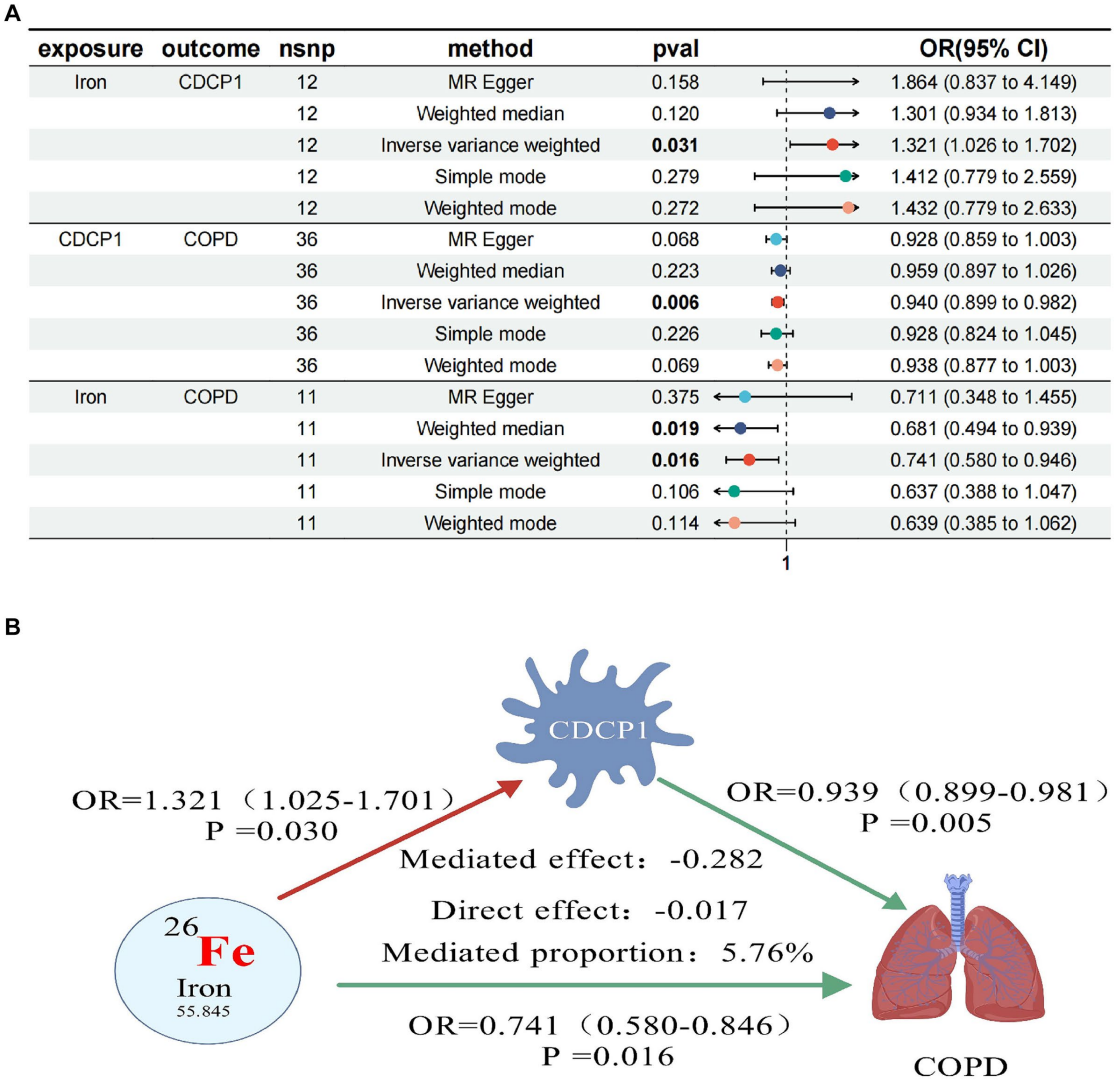
Forest plots of trace elements iron, CDCP1 and COPD **(A)**; CDCP1 mediates causal relationship between trace element iron and COPD (red is a risk factor, green is a protective factor) **(B)**.

In our final mediation analysis, we elucidated the causal effect proportions of four trace elements on COPD, mediated by seven inflammatory proteins. It was discovered that only CDCP1 mediated the impact of iron on COPD, with a mediation effect of −0.282, a direct effect of −0.017, and a mediation proportion of 5.76%. Regrettably, the other mediation effects were not established (Figure 3).

## 4 Discussion

This study provides genetic evidence supporting the causal relationships between trace elements such as Folate, Vitamin D, Vitamin B12, and Iron, and COPD in univariate MR analysis. After adjusting for BMI, further MVMR analysis revealed that Folate, Vitamin D, and Iron are independent risk factors for COPD. Finally, through TSMR and mediation analysis, CDCP1 is suggested to partially mediate the causal relationship between Iron and COPD. Our findings offer insights into dietary management and trace element supplementation for patients with COPD.

Malnutrition and trace element deficiencies are integral components of the rehabilitation process for patients with COPD, exhibiting a profound connection (61). Compared to healthy controls, COPD patients exhibit significantly reduced levels of Folate, presenting a novel therapeutic target for the treatment of COPD (62). Folate possesses antioxidative properties (63) and the capability to ameliorate endoplasmic reticulum stress (64), correlating positively with pulmonary function in COPD patients (65), thereby enhancing lung function (66) and alleviating respiratory distress (67). A reduction in Folate intake may lead to restricted airflow (68), whereas increasing Folate intake could potentially benefit pulmonary function (69). Folate may confer protective effects against acute lung injury by mitigating inflammatory responses (70). Serum Folate levels are positively correlated with lung function in elderly males (71) and are also associated with pulmonary function in children with asthma (72). However, supplementation with Folate does not influence changes in FEV1 (67), nor has a significant correlation been observed between serum Folate levels and lung function in females (65). These results present contradictions, and our MR analysis serves as a complement to observational studies and systematic reviews. Vitamin D plays a crucial role in both innate and adaptive immunity (73) and acts as a significant regulator in defending against pulmonary infectionss (74). It may also contribute to reducing mortality from respiratory diseases. Additionally (75), supplementation with Vitamin D alone can enhance lung function (5). Prospective studies have identified a correlation between lower Vitamin D levels and accelerated decline in lung function (76). Systematic reviews have concluded that Vitamin D supplementation can reduce the risk of respiratory infections (34) and enhance resistance to such infections (77). In COPD patients, the response to Vitamin D supplementation is diminished compared to healthy controls (78), and supplementation does not affect the muscular response to resistance training in COPD patients treated with Vitamin D_3_ (79). While some studies suggest that Vitamin D supplementation does not reduce the exacerbation rate of COPD (80), it is inversely related to inflammatory signaling in COPD (81). A deficiency in Vitamin D receptors may increase pulmonary inflammation (82), and Vitamin D may inhibit COPD-related pulmonary emphysema by maintaining the homeostasis and functionality of alveolar macrophages (83). Despite some contradictions in research concerning Vitamin D and COPD (84), our analyses using MR and MVMR have established a causal relationship between Vitamin D and COPD.

Vitamin B12, as a supplement in the rehabilitation of COPD patients, can regulate the secretion of NT-proBNP (85), exerting a positive effect on patients with advanced COPD (86). However, the intake of Vitamin B12 is not associated with the risk of frailty in COPD. After adjusting for BMI, our multivariate MR analysis indicates that Vitamin B12 is not an independent risk factor for COPD (87). Iron regulation is significantly associated with respiratory diseases (88). Dysregulation of iron homeostasis is a critical mechanism in lung injury (89). Iron-induced cell death can lead to airway remodeling and emphysema (90), exacerbating inflammation and oxidative stress (91). Targeting iron-induced cell death may ameliorate respiratory diseases (92) and alleviate the progression of COPD (93). Iron is related to the genetic susceptibility of COPD (94, 95), and COPD patients may experience non-anemic iron deficiency (96), which is associated with inflammatory responses (97), skeletal muscle disorders (98), hypoxemia, and reduced exercise tolerance (99). Clinical studies have shown that iron supplementation can improve the exercise endurance and quality of life of COPD patients (100, 101). Non-anemic iron deficiency can impair the response of COPD patients to pulmonary rehabilitation, resulting in lower aerobic capacity (102). Iron deficiency is linked to more severe pulmonary vascular diseases (103). Dysregulation of iron homeostasis in the lungs and cellular iron accumulation are factors in the development of COPD (104). Ferroptosis, an iron-dependent form of cell death, plays a role in the pathogenesis of COPD (105) and can ameliorate cigarette smoke-induced inflammation and emphysema (106). CXCL10 is a potential biomarker for impaired lung development (107), capable of modulating pulmonary inflammation (108) and the lung microenvironment (109). There is a correlation between EN-RAGE and COPD (110). CD6 serves as a therapeutic target in cancer immunotherapy (111), while CD40 is associated with the severity of COPD and the degree of pulmonary function alteration (112). Additionally, a correlation exists between CXCL6 and mortality in IPF (113). CDCP1, which may be involved in cell adhesion and matrix binding, could serve as a biomarker for lung cancer detection (114) and is somewhat associated with COVID-19 (115). Our research suggests that iron may mediate the effects on COPD through its influence on the inflammatory protein CDCP1, necessitating further exploration of the relationship between inflammatory responses, trace elements, and COPD.

This study, through MR analysis, investigates the causal relationships between trace elements, inflammatory proteins, and COPD, aiming to provide scientifically sound dietary recommendations for COPD patients and further suggest that supplementation with trace elements may be beneficial for COPD. This research has certain limitations; primarily, the study population is confined to Europeans, which may restrict the generalizability of the findings. Secondly, there is a need for a deeper exploration of the mechanisms linking trace elements, inflammatory proteins, and COPD, as the mediating effects observed were not significant, necessitating further.

## 5 Conclusion

In conclusion, our research demonstrates a causal relationship between genetically predicted trace elements such as Folate, Vitamin D, Vitamin B12, and Iron, and COPD. After adjusting for BMI, Folate, Vitamin D, and Iron emerge as independent risk factors for COPD. Furthermore, the inflammatory protein CDCP1 may play a partial mediating role in the causal relationship between Iron and COPD. Our findings can better inform scientifically sound dietary recommendations for patients, suggesting that supplementation with trace elements may be beneficial for those suffering from COPD.

## Data Availability

The original contributions presented in the study are included in the article/Supplementary material, further inquiries can be directed to the corresponding authors.

## Footnotes

^1^https://gwas.mrcieu.ac.uk/

^2^https://www.finngen.fi/en

## References

[1] WHO. Global health estimates: leading causes of death. Cause-specific mortality 2000–2019. Available at: https://www.who.int/data/gho/data/themes/mortality-and-global-health-estimates/ghe-leading-causes-of-death.

[2] Global Initiative for Chronic Obstructive Lung Disease (Gold). Global strategy for the diagnosis, management and prevention of chronic obstructive lung disease (2024 Report). Available at: https://goldcopd.org/.

[3] Keogh E Mark WE. Managing malnutrition in COPD: a review. Respir Med. (2021) 176:106248. doi: 10.1016/j.rmed.2020.106248, PMID: 33253970

[4] Fekete M Csipo T Fazekas-Pongor V Feher A Szarvas Z Kaposvari C. The effectiveness of supplementation with key vitamins, minerals, antioxidants and specific nutritional supplements in COPD-a review. Nutrients. (2023) 15:2741. doi: 10.3390/nu15122741, PMID: 37375645 PMC10300814

[5] Li M Zhao L Hu C Li Y Yang Y Zhang X. Improvement of lung function by micronutrient supplementation in patients with COPD: a systematic review and meta-analysis. Nutrients. (2024) 16:1028. doi: 10.3390/nu16071028, PMID: 38613061 PMC11013492

[6] Laudisio A Costanzo L Di Gioia C Delussu AS Traballesi M Gemma A. Dietary intake of elderly outpatients with chronic obstructive pulmonary disease. Arch Gerontol Geriatr. (2016) 64:75–81. doi: 10.1016/j.archger.2016.01.006, PMID: 26952380

[7] Nguyen HT Collins PF Pavey TG Nguyen NV Pham TD Gallegos DL. Nutritional status, dietary intake, and health-related quality of life in outpatients with COPD. Int J Chron Obstruct Pulmon Dis. (2019) 14:215–26. doi: 10.2147/COPD.S181322, PMID: 30666102 PMC6336029

[8] Zhai T Li S Hu W Li D Leng S. Potential micronutrients and phytochemicals against the pathogenesis of chronic obstructive pulmonary disease and lung cancer. Nutrients. (2018) 10:813. doi: 10.3390/nu10070813, PMID: 29941777 PMC6073117

[9] Collins PF Yang IA Chang YC Vaughan A. nutritional support in chronic obstructive pulmonary disease (COPD): an evidence update. J Thorac Dis. (2019) 11:S2230–7. doi: 10.21037/jtd.2019.10.41, PMID: 31737350 PMC6831917

[10] HuangWJFanXXYangYHZengYMKoCY. A review on the role of oral nutritional supplements in chronic obstructive pulmonary disease. J Nutr Health Aging. (2022) 26:723–31. doi: 10.1007/s12603-022-1822-835842763

[11] Beijers R Steiner MC Schols A. The role of diet and nutrition in the management of COPD. Eur Respir Rev. (2023) 32:230003. doi: 10.1183/16000617.0003-2023, PMID: 37286221 PMC10245132

[12] Whyand T Hurst JR Beckles M Caplin ME. Pollution and respiratory disease: can diet or supplements help? A review. Respir Res. (2018) 19:79. doi: 10.1186/s12931-018-0785-0, PMID: 29716592 PMC5930792

[13] Salari-Moghaddam A Milajerdi A Larijani B Esmaillzadeh A. Processed red meat intake and risk of COPD: a systematic review and dose-response meta-analysis of prospective cohort studies. Clin Nutr. (2019) 38:1109–16. doi: 10.1016/j.clnu.2018.05.020, PMID: 29909249

[14] Szmidt MK Kaluza J Harris HR Linden A Wolk A. Long-term dietary fiber intake and risk of chronic obstructive pulmonary disease: a prospective cohort study of women. Eur J Nutr. (2020) 59:1869–79. doi: 10.1007/s00394-019-02038-w, PMID: 31280344 PMC7351821

[15] Marco E Sanchez-Rodriguez D Davalos-Yerovi VN Duran X Pascual EM Muniesa JM. Malnutrition according to ESPEN consensus predicts hospitalizations and long-term mortality in rehabilitation patients with stable chronic obstructive pulmonary disease. Clin Nutr. (2019) 38:2180–6. doi: 10.1016/j.clnu.2018.09.014, PMID: 30342931

[16] Karim T Muhit M Khandaker G. Interventions to prevent respiratory diseases-nutrition and the developing world. Paediatr Respir Rev. (2017) 22:31–7. doi: 10.1016/j.prrv.2016.09.003 27793738

[17] Marin-Hinojosa C Eraso CC Sanchez-Lopez V Hernandez LC Otero-Candelera R Lopez-Campos JL. Nutriepigenomics and chronic obstructive pulmonary disease: potential role of dietary and epigenetics factors in disease development and management. Am J Clin Nutr. (2021) 114:1894–906. doi: 10.1093/ajcn/nqab267 34477827

[18] Cai X Song S Hu J Wang L Shen D Zhu Q. Systemic inflammation response index as a predictor of stroke risk in elderly patients with hypertension: a cohort study. J Inflamm Res. (2023) 16:4821–32. doi: 10.2147/JIR.S433190, PMID: 37901383 PMC10612501

[19] Ma H Cai X Hu J Song S Zhu Q Zhang Y. Association of systemic inflammatory response index with bone mineral density, osteoporosis, and future fracture risk in elderly hypertensive patients. Postgrad Med. (2024) 136:406–16. doi: 10.1080/00325481.2024.2354158, PMID: 38753519

[20] Guo Y Liu Q Zheng Z Qing M Yao T Wang B. Genetic association of inflammatory marker glyca with lung function and respiratory diseases. Nat Commun. (2024) 15:3751. doi: 10.1038/s41467-024-47845-w, PMID: 38704398 PMC11069551

[21] Scoditti E Massaro M Garbarino S Toraldo DM. Role of diet in chronic obstructive pulmonary disease prevention and treatment. Nutrients. (2019) 11:1357. doi: 10.3390/nu11061357 31208151 PMC6627281

[22] Cui Y Du X Li Y Wang D Lv Z Yuan H. Imbalanced and unchecked: the role of metal dyshomeostasis in driving COPD progression. COPD. (2024) 21:2322605. doi: 10.1080/15412555.2024.2322605, PMID: 38591165

[23] Han L Zhu W Qi H He L Wang Q Shen J. The cuproptosis-related gene glutaminase promotes alveolar macrophage copper ion accumulation in chronic obstructive pulmonary disease. Int Immunopharmacol. (2024) 129:111585. doi: 10.1016/j.intimp.2024.111585, PMID: 38325045

[24] Lenander-Ramirez A Bryngelsson IL Vihlborg P Westberg H Andersson L. Respirable dust and silica: respiratory diseases among Swedish iron foundry workers. J Occup Environ Med. (2022) 64:593–8. doi: 10.1097/JOM.0000000000002533, PMID: 35275887 PMC9301988

[25] Hua C Ma W Zheng F Zhang Y Xie J Ma L. Health risks and sources of trace elements and black carbon in Pm(2.5) from 2019 to 2021 in Beijing. J Environ Sci (China). (2024) 142:69–82. doi: 10.1016/j.jes.2023.05.023 38527897

[26] Wang Y Liao S Pan Z Jiang S Fan J Yu S. Hydrogen sulfide alleviates particulate matter-induced emphysema and airway inflammation by suppressing ferroptosis. Free Radic Biol Med. (2022) 186:1–16. doi: 10.1016/j.freeradbiomed.2022.04.014 35490984

[27] Jiang C Wu B Xue M Lin J Hu Z Nie X. Inflammation accelerates copper-mediated cytotoxicity through induction of six-transmembrane epithelial antigens of prostate 4 expression. Immunol Cell Biol. (2021) 99:392–402. doi: 10.1111/imcb.12427, PMID: 33179273

[28] Luan R Ding D Xue Q Li H Wang Y Yang J. Protective role of zinc in the pathogenesis of respiratory diseases. Eur J Clin Nutr. (2023) 77:427–35. doi: 10.1038/s41430-022-01191-6, PMID: 35982216 PMC9387421

[29] Liu X Ali MK Dua K Xu R. The role of zinc in the pathogenesis of lung disease. Nutrients. (2022) 14:2115. doi: 10.3390/nu14102115, PMID: 35631256 PMC9143957

[30] Shen T Bimali M Faramawi M Orloff MS. Consumption of vitamin K and vitamin a are associated with reduced risk of developing emphysema: Nhanes 2007-2016. Front Nutr. (2020) 7:47. doi: 10.3389/fnut.2020.00047, PMID: 32391372 PMC7192023

[31] Paulus MC Drent M Kouw IWK Balvers MGJ Bast A van Zanten ARH. Vitamin K: a potential missing link in critical illness-a scoping review. Crit Care. (2024) 28:212. doi: 10.1186/s13054-024-05001-2, PMID: 38956732 PMC11218309

[32] Thyagarajan B Meyer KA Smith LJ Beckett WS Williams OD Gross MD. Serum carotenoid concentrations predict lung function evolution in young adults: the coronary artery risk development in young adults (CARDIA) study. Am J Clin Nutr. (2011) 94:1211–8. doi: 10.3945/ajcn.111.019067, PMID: 21918220 PMC3192474

[33] Jun L Root M. Association of carotenoid intake with pulmonary function. J Am Coll Nutr. (2021) 40:708–12. doi: 10.1080/07315724.2020.1815608, PMID: 33030982

[34] Jolliffe DA Camargo CA Jr Sluyter JD Aglipay M Aloia JF Ganmaa D. Vitamin D supplementation to prevent acute respiratory infections: a systematic review and meta-analysis of aggregate data from randomised controlled trials. Lancet Diabetes Endocrinol. (2021) 9:276–92. doi: 10.1016/S2213-8587(21)00051-6, PMID: 33798465

[35] Liu Z Su Y Chen Q Xiao L Zhao X Wang F. Association of dietary intake of vitamin E with chronic obstructive pulmonary disease events in US adults: a cross-sectional study of Nhanes 2013-2018. Front Nutr. (2023) 10:1124648. doi: 10.3389/fnut.2023.1124648, PMID: 37125038 PMC10130507

[36] Lepp HL Amrein K Dizdar OS Casaer MP Gundogan K de Man AME. Lll 44- module 3: micronutrients in chronic disease. Clin Nutr ESPEN. (2024) 62:285–95. doi: 10.1016/j.clnesp.2024.05.009, PMID: 38875118

[37] Deng M Lu Y Zhang Q Bian Y Zhou X Hou G. Global prevalence of malnutrition in patients with chronic obstructive pulmonary disease: systemic review and meta-analysis. Clin Nutr. (2023) 42:848–58. doi: 10.1016/j.clnu.2023.04.005, PMID: 37084471

[38] Collins PF Stratton RJ Elia M. Nutritional support in chronic obstructive pulmonary disease: a systematic review and meta-analysis. Am J Clin Nutr. (2012) 95:1385–95. doi: 10.3945/ajcn.111.023499, PMID: 22513295

[39] Varraso R Camargo CA Jr. More evidence for the importance of nutritional factors in chronic obstructive pulmonary disease. Am J Clin Nutr. (2012) 95:1301–2. doi: 10.3945/ajcn.112.039834, PMID: 22552036

[40] Deutz NE Ziegler TR Matheson EM Matarese LE Tappenden KA Baggs GE. Reduced mortality risk in malnourished hospitalized older adult patients with COPD treated with a specialized oral nutritional supplement: sub-group analysis of the nourish study. Clin Nutr. (2021) 40:1388–95. doi: 10.1016/j.clnu.2020.08.031, PMID: 32921503

[41] Ter Beek L van der Vaart H Wempe JB Krijnen WP Roodenburg JLN van der Schans CP. Coexistence of malnutrition, frailty, physical frailty and disability in patients with COPD starting a pulmonary rehabilitation program. Clin Nutr. (2020) 39:2557–63. doi: 10.1016/j.clnu.2019.11.016, PMID: 31796229

[42] Emdin CA Khera AV Kathiresan S. Mendelian randomization. JAMA. (2017) 318:1925–6. doi: 10.1001/jama.2017.17219 29164242

[43] Skrivankova VW Richmond RC Woolf BAR Yarmolinsky J Davies NM Swanson SA. Strengthening the reporting of observational studies in epidemiology using Mendelian randomization: the strobe-MR Statement. JAMA. (2021) 326:1614–21. doi: 10.1001/jama.2021.18236, PMID: 34698778

[44] Zhao JH Stacey D Eriksson N Macdonald-Dunlop E Hedman AK Kalnapenkis A. Genetics of circulating inflammatory proteins identifies drivers of immune-mediated disease risk and therapeutic targets. Nat Immunol. (2023) 24:1540–51. doi: 10.1038/s41590-023-01588-w, PMID: 37563310 PMC10457199

[45] Kurki MI Karjalainen J Palta P Sipila TP Kristiansson K Donner KM. Finngen provides genetic insights from a well-phenotyped isolated population. Nature. (2023) 613:508–18. doi: 10.1038/s41586-022-05473-8, PMID: 36653562 PMC9849126

[46] Burgess S Scott RA Timpson NJ Davey Smith G Thompson SG Consortium E-I. Using published data in Mendelian randomization: a blueprint for efficient identification of causal risk factors. Eur J Epidemiol. (2015) 30:543–52. doi: 10.1007/s10654-015-0011-z, PMID: 25773750 PMC4516908

[47] Davies NM Holmes MV Davey SG. Reading Mendelian randomisation studies: a guide, glossary, and checklist for clinicians. BMJ. (2018) 362:k601. doi: 10.1136/bmj.k601, PMID: 30002074 PMC6041728

[48] He M Xu C Yang R Liu L Zhou D Yan S. Causal relationship between human blood metabolites and risk of ischemic stroke: a Mendelian randomization study. Front Genet. (2024) 15:1333454. doi: 10.3389/fgene.2024.1333454, PMID: 38313676 PMC10834680

[49] Yuan J Xiong X Zhang B Feng Q Zhang J Wang W. Genetically predicted C-reactive protein mediates the association between rheumatoid arthritis and atlantoaxial subluxation. Front Endocrinol (Lausanne). (2022) 13:1054206. doi: 10.3389/fendo.2022.1054206, PMID: 36589832 PMC9800511

[50] Choi KW Chen CY Stein MB Klimentidis YC Wang MJ Koenen KC. Assessment of bidirectional relationships between physical activity and depression among adults: a 2-sample mendelian randomization study. JAMA Psychiatry. (2019) 76:399–408. doi: 10.1001/jamapsychiatry.2018.4175, PMID: 30673066 PMC6450288

[51] Cheng ZX Hua JL Jie ZJ Li XJ Zhang J. Genetic insights into the gut-lung axis: mendelian randomization analysis on gut microbiota, lung function, and COPD. Int J Chron Obstruct Pulmon Dis. (2024) 19:643–53. doi: 10.2147/COPD.S441242, PMID: 38464560 PMC10921945

[52] Pierce BL Burgess S. Efficient design for Mendelian randomization studies: subsample and 2-sample instrumental variable estimators. Am J Epidemiol. (2013) 178:1177–84. doi: 10.1093/aje/kwt084, PMID: 23863760 PMC3783091

[53] Sanderson E. Multivariable Mendelian randomization and mediation. Cold Spring Harb Perspect Med. (2021) 11:a038984. doi: 10.1101/cshperspect.a038984, PMID: 32341063 PMC7849347

[54] Burgess S Thompson SG. Erratum to: interpreting findings from MENDELIAN randomization using the MR-Egger method. Eur J Epidemiol. (2017) 32:391–2. doi: 10.1007/s10654-017-0276-5, PMID: 28664250 PMC6828068

[55] Burgess S Bowden J Fall T Ingelsson E Thompson SG. Sensitivity analyses for robust causal inference from Mendelian randomization analyses with multiple genetic variants. Epidemiology. (2017) 28:30–42. doi: 10.1097/EDE.0000000000000559, PMID: 27749700 PMC5133381

[56] Bowden J Davey Smith G Burgess S. Mendelian randomization with invalid instruments: effect estimation and bias detection through Egger regression. Int J Epidemiol. (2015) 44:512–25. doi: 10.1093/ije/dyv080, PMID: 26050253 PMC4469799

[57] Verbanck M Chen CY Neale B Do R. Detection of widespread horizontal pleiotropy in causal relationships inferred from Mendelian randomization between complex traits and diseases. Nat Genet. (2018) 50:693–8. doi: 10.1038/s41588-018-0099-7, PMID: 29686387 PMC6083837

[58] Cai J Li X Wu S Tian Y Zhang Y Wei Z. Assessing the causal association between human blood metabolites and the risk of epilepsy. J Transl Med. (2022) 20:437. doi: 10.1186/s12967-022-03648-5, PMID: 36180952 PMC9524049

[59] Shi Y Feng S Yan M Wei S Yang K Feng Y. inflammatory bowel disease and celiac disease: a bidirectional Mendelian randomization study. Front Genet. (2022) 13:928944. doi: 10.3389/fgene.2022.928944, PMID: 36061176 PMC9437575

[60] Carter AR Sanderson E Hammerton G Richmond RC Davey Smith G Heron J. Mendelian randomisation for mediation analysis: current methods and challenges for implementation. Eur J Epidemiol. (2021) 36:465–78. doi: 10.1007/s10654-021-00757-1, PMID: 33961203 PMC8159796

[61] Rondanelli M Faliva MA Peroni G Infantino V Gasparri C Iannello G. Food pyramid for subjects with chronic obstructive pulmonary diseases. Int J Chron Obstruct Pulmon Dis. (2020) 15:1435–48. doi: 10.2147/COPD.S240561, PMID: 32606652 PMC7310971

[62] Zinellu A Mangoni AA. Arginine, transsulfuration, and folic acid pathway metabolomics in chronic obstructive pulmonary disease: a systematic review and meta-analysis. Cells. (2023) 12:2180. doi: 10.3390/cells12172180, PMID: 37681911 PMC10486395

[63] Cui S Lv X Li W Li Z Liu H Gao Y. Folic acid modulates VPO 1 DNA methylation levels and alleviates oxidative stress-induced apoptosis *in vivo* and *in vitro*. Redox Biol. (2018) 19:81–91. doi: 10.1016/j.redox.2018.08.005, PMID: 30125807 PMC6105767

[64] Nakano H Inoue S Minegishi Y Igarashi A Tokairin Y Yamauchi K. Effect of hyperhomocysteinemia on a murine model of smoke-induced pulmonary emphysema. Sci Rep. (2022) 12:12968. doi: 10.1038/s41598-022-16767-2, PMID: 35902671 PMC9334265

[65] Kim T Oak CH Jung MH Jang TW Kim J. High serum folate concentration is associated with better lung function in male chronic obstructive pulmonary disease patients who are current smokers: analysis of nationwide population-based survey. Nutrients. (2020) 12:2219. doi: 10.3390/nu12082219 32722447 PMC7468925

[66] Leng S Picchi MA Tesfaigzi Y Wu G Gauderman WJ Xu F. Dietary nutrients associated with preservation of lung function in Hispanic and non-Hispanic white smokers from New Mexico. Int J Chron Obstruct Pulmon Dis. (2017) 12:3171–81. doi: 10.2147/COPD.S142237, PMID: 29133979 PMC5669789

[67] Khan NA Saini H Mawari G Kumar S Hira HS Daga MK. The effect of folic acid supplementation on hyperhomocysteinemia and pulmonary function parameters in chronic obstructive pulmonary disease: a pilot study. J Clin Diagn Res. (2016) 10:OC17-OC21. doi: 10.7860/JCDR/2016/21322.8927, PMID: 28050421 PMC5198374

[68] Jung YJ Lee SH Chang JH Lee HS Kang EH Lee SW. The impact of changes in the intake of fiber and antioxidants on the development of chronic obstructive pulmonary disease. Nutrients. (2021) 13:580. doi: 10.3390/nu13020580, PMID: 33578669 PMC7916350

[69] Hirayama F Lee AH Terasawa K Kagawa Y. Folate intake associated with lung function, breathlessness and the prevalence of chronic obstructive pulmonary disease. Asia Pac J Clin Nutr. (2010) 19:103–9. PMID: 20199994

[70] Bai J Tang L Luo Y Han Z Li C Sun Y. Vitamin B complex blocks the dust fall Pm(2) (.5) -induced acute lung injury through DNA methylation in rats. Environ Toxicol. (2023) 38:403–14. doi: 10.1002/tox.23689, PMID: 36282901

[71] Chang SW Kim MB Kang JW. High serum folate level is positively associated with pulmonary function in elderly Korean men, but not in women. Sci Rep. (2022) 12:4523. doi: 10.1038/s41598-022-08234-9, PMID: 35296703 PMC8927119

[72] Papamichael MM Katsardis C Tsoukalas D Lambert K Erbas B Itsiopoulos C. Potential role of folate status on pulmonary function in pediatric asthma. Nutrition. (2021) 90:111267. doi: 10.1016/j.nut.2021.111267, PMID: 33979761

[73] Janssens W Decramer M Mathieu C Korf H. Vitamin D and chronic obstructive pulmonary disease: hype or reality? Lancet Respir Med. (2013) 1:804–12. doi: 10.1016/S2213-2600(13)70102-4 24461760

[74] Hiemstra PS de Jongh RT. Vitamin D deficiency in asthma and chronic obstructive pulmonary disease. a chicken-or-egg story. Am J Respir Crit Care Med. (2020) 202:312–3. doi: 10.1164/rccm.202004-1012ED, PMID: 32352312 PMC7397786

[75] Brenner H Holleczek B Schottker B. Vitamin D insufficiency and deficiency and mortality from respiratory diseases in a cohort of older adults: potential for limiting the death toll during and beyond the COVID-19 pandemic? Nutrients. (2020) 12:2488. doi: 10.3390/nu12082488, PMID: 32824839 PMC7468980

[76] Afzal S Lange P Bojesen SE Freiberg JJ Nordestgaard BG. Plasma 25-hydroxyvitamin D, lung function and risk of chronic obstructive pulmonary disease. Thorax. (2014) 69:24–31. doi: 10.1136/thoraxjnl-2013-203682, PMID: 23908128

[77] Anitua E Tierno R Alkhraisat MH. Current opinion on the role of vitamin d supplementation in respiratory infections and asthma/COPD exacerbations: a need to establish publication guidelines for overcoming the unpublished data. Clin Nutr. (2022) 41:755–77. doi: 10.1016/j.clnu.2022.01.029, PMID: 35182989

[78] Jolliffe DA Stefanidis C Wang Z Kermani NZ Dimitrov V White JH. Vitamin D metabolism is dysregulated in asthma and chronic obstructive pulmonary disease. Am J Respir Crit Care Med. (2020) 202:371–82. doi: 10.1164/rccm.201909-1867OC, PMID: 32186892 PMC7397796

[79] Molmen KS Hammarstrom D Pedersen K Lian Lie AC Steile RB Nygaard H. Vitamin D (3) supplementation does not enhance the effects of resistance training in older adults. J Cachexia Sarcopenia Muscle. (2021) 12:599–628. doi: 10.1002/jcsm.12688, PMID: 33788419 PMC8200443

[80] Rafiq R Aleva FE Schrumpf JA Daniels JM Bet PM Boersma WG. Vitamin D supplementation in chronic obstructive pulmonary disease patients with low serum vitamin D: a randomized controlled trial. Am J Clin Nutr. (2022) 116:491–9. doi: 10.1093/ajcn/nqac083, PMID: 35383823 PMC9348978

[81] Fu L Fei J Tan ZX Chen YH Hu B Xiang HX. Low vitamin D status is associated with inflammation in patients with chronic obstructive pulmonary disease. J Immunol. (2021) 206:515–23. doi: 10.4049/jimmunol.2000964, PMID: 33361208 PMC7812059

[82] Sundar IK Hwang JW Wu S Sun J Rahman I. Deletion of vitamin D receptor leads to premature emphysema/COPD by increased matrix metalloproteinases and lymphoid aggregates formation. Biochem Biophys Res Commun. (2011) 406:127–33. doi: 10.1016/j.bbrc.2011.02.011, PMID: 21300024 PMC3049841

[83] Hu G Dong T Wang S Jing H Chen J. Vitamin D (3)-vitamin D receptor axis suppresses pulmonary emphysema by maintaining alveolar macrophage homeostasis and function. EBioMedicine. (2019) 45:563–77. doi: 10.1016/j.ebiom.2019.06.039, PMID: 31278070 PMC6642288

[84] Jolliffe DA Greenberg L Hooper RL Mathyssen C Rafiq R de Jongh RT. Vitamin D to prevent exacerbations of COPD: systematic review and meta-analysis of individual participant data from randomised controlled trials. Thorax. (2019) 74:337–45. doi: 10.1136/thoraxjnl-2018-212092 30630893

[85] Paulin FV Goelzer LS Muller PT. Vitamin B(12) supplementation and Nt-Probnp levels in COPD patients: a secondary analysis of a randomized and controlled study in rehabilitation. Front Neurosci. (2020) 14:740. doi: 10.3389/fnins.2020.00740, PMID: 32760247 PMC7372128

[86] Paulin FV Zagatto AM Chiappa GR Muller PT. Addition of vitamin B12 to exercise training improves cycle ergometer endurance in advanced COPD patients: a randomized and controlled study. Respir Med. (2017) 122:23–9. doi: 10.1016/j.rmed.2016.11.015, PMID: 27993287

[87] Cheng X Hu Y Ruan Z Zang G Chen X Qiu Z. Association between B-vitamins intake and frailty among patients with chronic obstructive pulmonary disease. Aging Clin Exp Res. (2023) 35:793–801. doi: 10.1007/s40520-023-02353-7, PMID: 36719551

[88] Ali MK Kim RY Brown AC Mayall JR Karim R Pinkerton JW. Crucial role for lung iron level and regulation in the pathogenesis and severity of asthma. Eur Respir J. (2020) 55:1901340. doi: 10.1183/13993003.01340-2019, PMID: 32184317

[89] Kang H Huang D Zhang W Wang J Liu Z Wang Z. Inhaled polystyrene microplastics impaired lung function through pulmonary flora/Tlr 4-mediated iron homeostasis imbalance. Sci Total Environ. (2024) 946:174300. doi: 10.1016/j.scitotenv.2024.174300, PMID: 38936707

[90] Xia H Wu Y Zhao J Cheng C Lin J Yang Y. N6-Methyladenosine-Modified Circsav 1 triggers ferroptosis in Copd through recruiting Ythdf1 to facilitate the translation of Ireb2. Cell Death Differ. (2023) 30:1293–304. doi: 10.1038/s41418-023-01138-9, PMID: 36828914 PMC10154389

[91] Galli TT de Campos EC do Nascimento Camargo L Fukuzaki S Dos Santos TM SSS H. Effects of environmental exposure to iron powder on healthy and elastase-exposed mice. Sci Rep. (2024) 14:9134. doi: 10.1038/s41598-024-59573-8, PMID: 38644380 PMC11033283

[92] Xu M Zhang D Yan J. Targeting ferroptosis using Chinese herbal compounds to treat respiratory diseases. Phytomedicine. (2024) 130:155738. doi: 10.1016/j.phymed.2024.155738, PMID: 38824825

[93] Liu L Zhang Y Wang L Liu Y Chen H Hu Q. Scutellarein alleviates chronic obstructive pulmonary disease through inhibition of ferroptosis by chelating iron and interacting with arachidonate 15-lipoxygenase. Phytother Res. (2023) 37:4587–606. doi: 10.1002/ptr.7928, PMID: 37353982

[94] Cloonan SM Mumby S Adcock IM Choi AMK Chung KF Quinlan GJ. The "Iron"-Y of iron overload and iron deficiency in chronic obstructive pulmonary disease. Am J Respir Crit Care Med. (2017) 196:1103–12. doi: 10.1164/rccm.201702-0311PP, PMID: 28410559 PMC5694836

[95] Mariani TJ. Respiratory disorders: ironing out smoking-related airway disease. Nature. (2016) 531:586–7. doi: 10.1038/nature17309 26958829

[96] Perez-Peiro M Martin-Ontiyuelo C Rodo-Pi A Piccari L Admetllo M Duran X. Iron replacement and redox balance in non-anemic and mildly anemic iron deficiency COPD patients: insights from a clinical trial. Biomedicines. (2021) 9:1191. doi: 10.3390/biomedicines9091191, PMID: 34572377 PMC8470868

[97] Verhamme FM De Smet EG Van Hooste W Delanghe J Verleden SE Joos GF. Bone morphogenetic protein 6 (BMP-6) modulates lung function, pulmonary iron levels and cigarette smoke-induced inflammation. Mucosal Immunol. (2019) 12:340–51. doi: 10.1038/s41385-018-0116-2, PMID: 30542109

[98] Dziegala M Josiak K Kasztura M Kobak K von Haehling S Banasiak W. Iron deficiency as energetic insult to skeletal muscle in chronic diseases. J Cachexia Sarcopenia Muscle. (2018) 9:802–15. doi: 10.1002/jcsm.12314, PMID: 30178922 PMC6204587

[99] Nickol AH Frise MC Cheng HY McGahey A McFadyen BM Harris-Wright T. A cross-sectional study of the prevalence and associations of iron deficiency in a cohort of patients with chronic obstructive pulmonary disease. BMJ Open. (2015) 5:e007911. doi: 10.1136/bmjopen-2015-007911, PMID: 26150144 PMC4499677

[100] Martin-Ontiyuelo C Rodo-Pin A Echeverria-Esnal D Admetllo M Duran-Jorda X Alvarado M. Intravenous iron replacement improves exercise tolerance in COPD: a single-blind randomized trial. Arch Bronconeumol. (2022) 58:689–98. doi: 10.1016/j.arbres.2021.08.011, PMID: 35312562

[101] Grasmuk-Siegl E Urban MH Scherrer S Funk GC. Effect of intravenous ferric carboxymaltose on exercise capacity and quality of life in patients with COPD: a pilot study. Wien Klin Wochenschr. (2023) 135:35–44. doi: 10.1007/s00508-022-02073-4, PMID: 36044093

[102] Barberan-Garcia A Rodriguez DA Blanco I Gea J Torralba Y Arbillaga-Etxarri A. Non-anaemic iron deficiency impairs response to pulmonary rehabilitation in COPD. Respirology. (2015) 20:1089–95. doi: 10.1111/resp.12591, PMID: 26148453

[103] Tatah J Keen JL Prisco SZ Pritzker M Thenappan T Prins KW. Iron deficiency is associated with more severe pulmonary vascular disease in pulmonary hypertension caused by chronic lung disease. Chest. (2022) 161:232–6. doi: 10.1016/j.chest.2021.07.2159, PMID: 34352277 PMC8783028

[104] Faherty L Kenny S Cloonan SM. Iron and mitochondria in the susceptibility, pathogenesis and progression of COPD. Clin Sci (Lond). (2023) 137:219–37. doi: 10.1042/CS20210504, PMID: 36729089

[105] Yoshida M Minagawa S Araya J Sakamoto T Hara H Tsubouchi K. Involvement of cigarette smoke-induced epithelial cell ferroptosis in COPD pathogenesis. Nat Commun. (2019) 10:3145. doi: 10.1038/s41467-019-10991-7, PMID: 31316058 PMC6637122

[106] Cloonan SM Glass K Laucho-Contreras ME Bhashyam AR Cervo M Pabon MA. Mitochondrial iron chelation ameliorates cigarette smoke-induced bronchitis and emphysema in mice. Nat Med. (2016) 22:163–74. doi: 10.1038/nm.4021, PMID: 26752519 PMC4742374

[107] Wang G Hallberg J Faner R Koefoed HJ Kebede Merid S Klevebro S. Plasticity of individual lung function states from childhood to adulthood. Am J Respir Crit Care Med. (2023) 207:406–15. doi: 10.1164/rccm.202203-0444OC, PMID: 36409973 PMC9940138

[108] Hu L Yamamoto M Chen J Duan H Du J He L. Integrating network pharmacology and experimental verification to decipher the immunomodulatory effect of Bu-Zhong-Yi-Qi-Tang against Poly (I: C)-induced pulmonary inflammation. Front Pharmacol. (2022) 13:1015486. doi: 10.3389/fphar.2022.1015486, PMID: 36304166 PMC9592993

[109] Li Q Sun J Cao Y Liu B Zhao Z Hu L. Icaritin inhibited cigarette smoke extract-induced Cd8(+) T cell chemotaxis enhancement by targeting the Cxcl 10/Cxcr3 axis and Tgf-Beta/Smad 2 signaling. Phytomedicine. (2022) 96:153907. doi: 10.1016/j.phymed.2021.153907 35026517

[110] Lu T Lahousse L Wijnant S Chen J Brusselle GG van Hoek M. The age-rage axis associates with chronic pulmonary diseases and smoking in the rotterdam study. Respir Res. (2024) 25:85. doi: 10.1186/s12931-024-02698-1, PMID: 38336742 PMC10858545

[111] Ruth JH Gurrea-Rubio M Athukorala KS Rasmussen SM Weber DP Randon PM. Cd6 is a target for cancer immunotherapy. JCI Insight. (2021) 6:e145662. doi: 10.1172/jci.insight.145662, PMID: 33497367 PMC8021120

[112] Sun D Lin R Ouyang Y. The role of Cd40, Cd86, and glutathione S-transferase omega 1 in the pathogenesis of chronic obstructive pulmonary disease. Can Respir J. (2022) 2022:6810745. doi: 10.1155/2022/6810745, PMID: 36051533 PMC9427324

[113] Bahudhanapati H Tan J Apel RM Seeliger B Schupp J Li X. Increased Expression of Cxcl 6 in secretory cells drives fibroblast collagen synthesis and is associated with increased mortality in idiopathic pulmonary fibrosis. Eur Respir J. (2024) 63:2300088. doi: 10.1183/13993003.00088-2023, PMID: 37918852

[114] von Itzstein MS Gerber DE Minna JD. Contemporary lung cancer screening and the promise of blood-based biomarkers. Cancer Res. (2021) 81:3441–3. doi: 10.1158/0008-5472.CAN-21-0706, PMID: 34252039

[115] Baranova A Luo J Fu L Yao G Zhang F. Evaluating the effects of circulating inflammatory proteins as drivers and therapeutic targets for severe COVID-19. Front Immunol. (2024) 15:1352583. doi: 10.3389/fimmu.2024.1352583, PMID: 38455043 PMC10917991

