# Supplementary material for: Genetic information supports a causal relationship between trace elements, inflammatory proteins, and COPD: evidence from a Mendelian randomization analysis
Source: Front Nutr. 2024 Aug 14;11:1430606. doi: 10.3389/fnut.2024.1430606 (PMC11349556; doi:10.3389/fnut.2024.1430606)
Supplement: Supplementary file 1 [file Data_Sheet_1.PDF]

STROBE-MR checklist of recommended items to address in reports of Mendelian randomization studies[1 2]

Item No.	Section		Checklist item	Relevant text from manuscript
1	**TITLE and ABSTRACT**		Indicate Mendelian randomization (MR) as the study's design in the title and/or the abstract if that is a main purpose of the study	Genetic information supports a causal relationship between trace elements, inflammatory proteins, and COPD ： Evidence from a Mendelian randomization analysis
	**INTRODUCTION**			
2	**Background**		Explain the scientific background and rationale for the reported study. What is the exposure? Is a potential causal relationship between exposure and outcome plausible? Justify why MR is a helpful method to address the study question	Dietary factors and nutritional status may be among the risk factors for COPD. There exists a certain correlation between trace elements and COPD. Research has identified that diet can influence the onset of COPD through three primary mechanisms, with inflammation modulation being the most significant.
3	**Objectives**		State specific objectives clearly, including pre-specified causal hypotheses (if any). State that MR is a method that, under specific assumptions, intends to estimate causal effects	Through Mendelian Randomization (MR) analysis, we investigated the causal relationships between trace elements, inflammatory proteins, and COPD.
	**METHODS**			
4	**Study design and data sources**		Present key elements of the study design early in the article. Consider including a table listing sources of data for all phases of the study. For each data source contributing to the analysis, describe the following:	
		a)	Setting: Describe the study design and the underlying population, if possible. Describe the setting, locations, and relevant dates, including periods of recruitment, exposure, follow-up, and data collection, when available.	The genetic information for the 15 trace elements is sourced from the GWAS database (https://gwas.mrcieu.ac.uk/), all pertaining to European populations. The data for the 91 inflammatory proteins are derived from a 2023 study involving 14,824 Europeans,cataloged under the identifiers GCST90274001 to GCST90275000. The COPD data is obtained from the tenth round of analysis by the FinnGen database (https://www.finngen.fi/en), also concerning European populations. Additionally, the genetic information for BMI is acquired from the GWAS database and is likewise representative of European demographics.
		b)	Participants: Give the eligibility criteria, and the sources and methods of selection of participants. Report the sample size, and whether any power or sample size calculations were carried out prior to the main analysis	Table 1.
		c)	Describe measurement, quality control and selection of genetic variants	To ensure their relevance, we conduct an association analysis on the 15 trace elements using a significance threshold of P<5 × 10 − 6. Subsequently, we eliminate any single

				nucleotide polymorphisms (SNPs) exhibiting linkage disequilibrium by applying criteria of R2<0.001 and a kilobase range of 10,000. We then calculate the F-statistic for the selected SNPs to exclude weak instrumental variables, considering an F-value greater than 10 as indicative of the absence of weak instrumental variables.
		d)	For each exposure, outcome, and other relevant variables, describe methods of assessment and diagnostic criteria for diseases	Not Applicable
		e)	Provide details of ethics committee approval and participant informed consent, if relevant	Not Applicable
5	**Assumptions**		Explicitly state the three core IV assumptions for the main analysis (relevance, independence and exclusion restriction) as well assumptions for any additional or sensitivity analysis	The selection of instrumental variables must satisfy several assumptions: the instrumental variables should be closely associated with COPD, independent of confounding factors in the exposure-outcome relationship, and must influence COPD solely through the trace elements.
6	**Statistical methods: main analysis**		Describe statistical methods and statistics used	
		a)	Describe how quantitative variables were handled in the analyses (i.e., scale, units, model)	This research does not involve any transformations of quantitative variables.
		b)	Describe how genetic variants were handled in the analyses and, if applicable, how their weights were selected	To evaluate the causality, we employ five methods: Inverse Variance Weighted (IVW), MR-Egger, Weighted Median, Simple Mode, and Weighted Mode methods, with IVW serving as the primary method. A p-value of less than 0.05 indicates a causal relationship while the other four methods serve as supplementary approaches.
		c)	Describe the MR estimator (e.g. two-stage least squares, Wald ratio) and related statistics. Detail the included covariates and, in case of two-sample MR, whether the same covariate set was used for adjustment in the two samples	Genetic associations with all exposures were taken from a large meta-analysis of GWAS, we obtained SNP-specific Wald estimates and then used inverse variance weighting (IVW) with multiplicative random effects, MR-Egger, and weighted median (WM). The IVW method is a classical method for MR analysis, where the weighted average is calculated by taking the reciprocal of the variance of each IV as the weight, ensuring the effectiveness of all IVs. MR-Egger utilizes a weighted linear regression analysis, providing robust estimates that are independent of the validity of instrumental variables. Nevertheless, it is crucial to acknowledge that these estimates may have lower statistical precision and can be influenced by outlier genetic variation. On the other hand, The problem of estimation accuracy variability is tackled by the WM approach. In a

				manner reminiscent of the IVW approach, the WM method assigns inverse weights that are contingent upon the variance of individual genetic variants, demonstrating reliability even when causal effects are violated.
		d)	Explain how missing data were addressed	In this MR analysis, the issue of missing data was not involved.
		e)	If applicable, indicate how multiple testing was addressed	In this MR analysis, multiple exposures or multiple outcomes were not involved, so multiple testing was not performed.
7	**Assessment of assumptions**		Describe any methods or prior knowledge used to assess the assumptions or justify their validity	To evaluate the robustness of our results, we conducted sensitivity analysis using the "leave-one-out" technique. Additionally, we employed Cochran's Q test, MR-Egger intercept test, and MR-PRESSO to test for pleiotropy and heterogeneity, with a P-value greater than 0.05 indicating the absence of both.
8	**Sensitivity analyses and additional analyses**		Describe any sensitivity analyses or additional analyses performed (e.g. comparison of effect estimates from different approaches, independent replication, bias analytic techniques, validation of instruments, simulations)	To assess the robustness of our results, leave-one-out sensitivity analysis is conducted, further complemented by tests for pleiotropy and heterogeneity, with a p-value greater than 0.05 indicating the absence of both pleiotropy and heterogeneity. All analyses are performed using the R programming language (version 4.3.3).
9	**Software and pre-registration**			
		a)	Name statistical software and package(s), including version and settings used	All analyses were conducted using R version 4.3.3, with the software packages 'Two-SampleMR'. To visualize the MR analysis, forest plots, scatter plots, and leave-one-out plots were generated using the data analysis function of the Rstudio platform.
		b)	State whether the study protocol and details were pre-registered (as well as when and where)	This study was not pre-registered with the study protocol and details.
	**RESULTS**			
10	**Descriptive data**			
		a)	Report the numbers of individuals at each stage of included studies and reasons for exclusion. Consider use of a flow diagram	The genetic information for the 15 trace elements is sourced from the GWAS database (https://gwas.mrcieu.ac.uk/), all pertaining to European populations. The data for the 91 inflammatory proteins are derived from a 2023 study involving 14,824 Europeans,cataloged under the identifiers GCST90274001 to GCST90275000. The COPD data is

				obtained from the tenth round of analysis by the FinnGen database (https://www.finngen.fi/en), also concerning European populations. Additionally, the genetic information for BMI is acquired from the GWAS database and is likewise representative of European demographics.
		b)	Report summary statistics for phenotypic exposure(s), outcome(s), and other relevant variables (e.g. means, SDs, proportions)	Table 2,Table 3,Table 4, and Table 5.
		c)	If the data sources include meta-analyses of previous studies, provide the assessments of heterogeneity across these studies	Table 2,Table 3,Table 4, and Table 5.
		d)	For two-sample MR:<br>i. Provide justification of the similarity of the genetic variant-exposure associations between the exposure and outcome samples<br>ii. Provide information on the number of individuals who overlap between the exposure and outcome studies	The data presented in this study were derived exclusively from European population samples. These samples were obtained from independent GWAS databases, ensuring minimal overlap and bias, detailed data on the number of individuals in the exposure and outcome samples are provided in Table 2,Table 3,Table 4, and Table 5..
11	**Main results**			
		a)	Report the associations between genetic variant and exposure, and between genetic variant and outcome, preferably on an interpretable scale	Univariate MR analysis supports a causal relationship between trace elements such as Folate, Vitamin D, Vitamin B12, and Iron, and COPD. The results of the IVW analysis indicate a positive correlation between Folate (OR=1.293, 95% CI 1.027-1.628; P=0.029), Vitamin D (OR=1.331, 95% CI 1.071-1.654; P=0.010), and Vitamin B12 (OR=1.424, 95% CI 1.108-1.828; P=0.006) with COPD, while Iron shows a negative correlation (OR=0.741, 95% CI 0.580-0.946; P=0.016). Concurrently, reverse MR analysis revealed no reverse causality between Folate, Vitamin D, Vitamin B12, and Iron with COPD (P>0.05).
		b)	Report MR estimates of the relationship between exposure and outcome, and the measures of uncertainty from the MR analysis, on an interpretable scale, such as odds ratio or relative risk per SD difference	Table 2,Table 3,Table 4, and Table 5.
		c)	If relevant, consider translating estimates of relative risk into absolute risk for a meaningful time period	Not Applicable.
		d)	Consider plots to visualize results (e.g. forest plot, scatterplot of associations between genetic variants and outcome versus between genetic variants and exposure)	Figure 2 and Figure 3.
12	**Assessment of assumptions**			
		a)	Report the assessment of the validity of the assumptions	To evaluate the robustness of our analytical results, we

				employed Cochran's Q test, the MR-Egger intercept test, and MR-PRESSO to examine pleiotropy and heterogeneity. No evidence of pleiotropy or heterogeneity was detected (P>0.05). The leave-one-out analysis indicated that the exclusion of any single SNP would not significantly affect the estimation of causal relationships, suggesting that the results of the MR analysis are robust .
		b)	Report any additional statistics (e.g., assessments of heterogeneity across genetic variants, such as I^2, Q statistic or E-value)	Table 2,Table 3,Table 4, and Table 5.
13	**Sensitivity analyses and additional analyses**			
		a)	Report any sensitivity analyses to assess the robustness of the main results to violations of the assumptions	Table 2,Table 3,Table 4, and Table 5.
		b)	Report results from other sensitivity analyses or additional analyses	Table 2,Table 3,Table 4, and Table 5.
		c)	Report any assessment of direction of causal relationship (e.g., bidirectional MR)	Concurrently, reverse MR analysis revealed no reverse causality between Folate, Vitamin D, Vitamin B12, and Iron with COPD (P>0.05).
		d)	When relevant, report and compare with estimates from non-MR analyses	This study does not involve non-MR studies.
		e)	Consider additional plots to visualize results (e.g., leave-one-out analyses)	Figure 2
	**DISCUSSION**			
14	**Key results**		Summarize key results with reference to study objectives	Trace elements such as Folate (OR=1.293, 95%CI 1.027-1.628; P=0.029), Vitamin D (OR=1.331, 95%CI 1.071-1.654; P=0.010), Vitamin B12 (OR=1.424, 95%CI 1.108-1.828; P=0.006), and Iron (OR=0.741, 95%CI 0.580-0.946; P=0.016) demonstrated causal relationships with COPD. No causal relationship was observed in reverse MR. After adjusting for BMI, Folate (OR=1.633, 95%CI 1.098-2.429; P=0.015), Iron (OR=0.507, 95%CI 0.31-0.778; P=0.001), and Vitamin D (OR=1.511, 95%CI 1.029-2.217; P=0.034) were identified as independent risk factors for COPD, whereas Vitamin B12 (OR=1.118, 95%CI 0.751-1.666; P=0.581) was not. Mediation analysis indicated that CDCP1 (5.76%) may play a mediating role between Iron and COPD.
15	**Limitations**		Discuss limitations of the study, taking into account the validity of the IV assumptions, other sources of potential bias, and imprecision. Discuss both direction and	Firstly, the databases used in the study only included individuals of European ancestry. To obtain stronger

			magnitude of any potential bias and any efforts to address them	evidence, it is necessary to expand the databases to include other ethnic groups such as those from Asia and Africa. Secondly, the threshold of P value was less than 5× 10-8 is generally considered to indicate genome-wide significance when screening for IVs. However, in this study, the threshold of P value was set less than 5×10-6 in order to obtain a sufficient number of SNPs. It is important to interpret the study results with caution, as this difference in threshold may have some impact on the findings. Third, the MR analysis method is a theoretical causal analysis method that requires further validation through animal experiments to establish the causal relationship.
16	**Interpretation**			
		a)	Meaning: Give a cautious overall interpretation of results in the context of their limitations and in comparison with other studies	This research has certain limitations; primarily, the study population is confined to Europeans, which may restrict the generalizability of the findings. Secondly, there is a need for a deeper exploration of the mechanisms linking trace elements, inflammatory proteins, and COPD, as the mediating effects observed were not significant, necessitating further
		b)	Mechanism: Discuss underlying biological mechanisms that could drive a potential causal relationship between the investigated exposure and the outcome, and whether the gene-environment equivalence assumption is reasonable. Use causal language carefully, clarifying that IV estimates may provide causal effects only under certain assumptions	In conclusion, our research demonstrates a causal relationship between genetically predicted trace elements such as Folate, Vitamin D, Vitamin B12, and Iron, and COPD. After adjusting for BMI, Folate, Vitamin D, and Iron emerge as independent risk factors for COPD. Furthermore, the inflammatory protein CDCP1 may play a partial mediating role in the causal relationship between Iron and COPD.
		c)	Clinical relevance: Discuss whether the results have clinical or public policy relevance, and to what extent they inform effect sizes of possible interventions	Our findings can better inform scientifically sound dietary recommendations for patients, suggesting that supplementation with trace elements may be beneficial for those suffering from COPD.
17	**Generalizability**		Discuss the generalizability of the study results (a) to other populations, (b) across other exposure periods/timings, and (c) across other levels of exposure	However, it did not investigate the effects of varying exposure periods or levels. Furthermore, the study was limited to a European population, raising questions about its generalizability to other populations.
	**OTHER INFORMATION**			
18	**Funding**		Describe sources of funding and the role of funders in the present study and, if applicable, sources of funding for the databases and original study or studies on which the present study is based	The author(s) received no specific funding for this work.

19	**Data and data sharing**	Provide the data used to perform all analyses or report where and how the data can be accessed, and reference these sources in the article. Provide the statistical code needed to reproduce the results in the article, or report whether the code is publicly accessible and if so, where	We want to acknowledge the participants and investigators of the FinnGen study and GWAS, as well as all the authors for their contributions to this article.
20	**Conflicts of Interest**	All authors should declare all potential conflicts of interest	The authors declare that the research was conducted in the absence of any commercial or financial relationships that could be construed as potential conflicts of interest.

This checklist is copyrighted by the Equator Network under the Creative Commons Attribution 3.0 Unported (CC BY 3.0) license.

1. Skrivankova VW, Richmond RC, Woolf BAR, Yarmolinsky J, Davies NM, Swanson SA, et al. Strengthening the Reporting of Observational Studies in Epidemiology using Mendelian Randomization (STROBE-MR) Statement. JAMA. 2021;under review.

2. Skrivankova VW, Richmond RC, Woolf BAR, Davies NM, Swanson SA, VanderWeele TJ, et al. Strengthening the Reporting of Observational Studies in Epidemiology using Mendelian Randomisation (STROBE-MR): Explanation and Elaboration. BMJ. 2021;375:n2233.
